# Risk factors for comorbid oppositional defiant disorder in attention-deficit/hyperactivity disorder

**DOI:** 10.1007/s00787-017-0972-4

**Published:** 2017-03-10

**Authors:** Siri D. S. Noordermeer, Marjolein Luman, Wouter D. Weeda, Jan K. Buitelaar, Jennifer S. Richards, Catharina A. Hartman, Pieter J. Hoekstra, Barbara Franke, Dirk J. Heslenfeld, Jaap Oosterlaan

**Affiliations:** 1 Clinical Neuropsychology Section, Vrije Universiteit Amsterdam, Van der Boechorststraat 1, 1081 BT, Amsterdam, The Netherlands; 2 Leiden University, Leiden, The Netherlands; 3 Radboud University Medical Center, Nijmegen, The Netherlands; 4 Karakter Child and Adolescent Psychiatry University Centre, Nijmegen, The Netherlands; 5 University of Groningen, Groningen, The Netherlands

**Keywords:** ADHD, ODD, Risk factors, Comorbidity

## Abstract

Oppositional defiant disorder (ODD) is highly prevalent in attention-deficit/hyperactivity disorder (ADHD). Individuals with both ADHD and ODD (ADHD + ODD) show a considerably worse prognosis compared with individuals with either ADHD or ODD. Therefore, identification of risk factors for ADHD + ODD is essential and may contribute to the development of (early) preventive interventions. Participants were matched for age, gender, and ADHD-subtype (diagnostic groups), and did not differ in IQ. Predictors included pre- and perinatal risk factors (pregnancy duration, birth weight, maternal smoking during pregnancy), transgenerational factors (parental ADHD; parental warmth and criticism in diagnostic groups), and postnatal risk factors (parental socioeconomic status [SES], adverse life events, deviant peer affiliation). Three models were assessed, investigating risk factors for ADHD-only versus controls (*N* = 86), ADHD + ODD versus controls (*N* = 86), and ADHD + ODD versus ADHD-only (*N* = 90). Adverse life events and parental ADHD were risk factors for both ADHD + ODD and ADHD-only, and more adverse life events were an even stronger risk factor for comorbid ODD compared with ADHD-only. For ADHD + ODD, but not ADHD-only, parental criticism, deviant peer affiliation, and parental SES acted as risk factors. Maternal smoking during pregnancy acted as minor risk factor for ADHD-only, while higher birth weight acted as minor risk factor for ADHD + ODD. No effects of age were present. Findings emphasise the importance of these factors in the development of comorbid ODD. The identified risk factors may prove to be essential in preventive interventions for comorbid ODD in ADHD, highlighting the need for parent-focused interventions to take these factors into account.

**Electronic supplementary material:**

The online version of this article (doi:10.1007/s00787-017-0972-4) contains supplementary material, which is available to authorized users.

## Introduction

Attention-deficit/hyperactivity disorder (ADHD) is a common childhood psychiatric disorder that is defined by developmentally inappropriate levels of inattention, and/or hyperactivity and impulsivity [2]. Previously identified risk factors for ADHD include both genetic and environmental factors. However, the genetic risk factors identified thus far explain only a small percentage of the heritability [for extensive reviews see: 27, 50]. Environmental risk factors for ADHD have shown to have more substantial effect sizes than genetic factors [27], and may have more immediate relevance for clinical treatment. While some environmental factors can be used to provide early identification of at-risk individuals, others can possibly be counteracted by education, training, or interventions. Environmental factors can be subdivided into pre- and perinatal factors, transgenerational influences and postnatal factors. Well-documented pre- and perinatal factors for ADHD include premature birth, low birth weight and maternal smoking during pregnancy [32]. Low birth weight is one of the most investigated and consistently reported risk factors for ADHD, and might even (partly) explain the association between maternal smoking during pregnancy and ADHD [39, 41]. Well-documented transgenerational influences and postnatal risk factors include a family history of ADHD and higher levels of family conflict [36, 49], although a family history of ADHD is likely to comprise both environmental and genetic influences.

A condition which is frequently comorbid with ADHD is oppositional defiant disorder (ODD), occurring in up to 60% of individuals with ADHD [14, 21]. ODD is defined by a frequent and persistent pattern of irritable and angry mood, vindictiveness, and developmentally inappropriate, negativistic, defiant, and disobedient behaviour toward authority figures [2]. Individuals with both ADHD and ODD have a considerably worse prognosis than individuals with either one of the disorders in terms of an increased risk to develop anxiety and depressive disorders as well as conduct disorder and even antisocial personality disorder later in life [4, 35]. Furthermore, the comorbid group shows an earlier onset with more functional impairments and exhibits more physical aggression and delinquency than individuals with ADHD or ODD alone [4, 34, 35]. This emphasises the need to not only identify risk factors for ADHD, but especially for ODD comorbid with ADHD. The identification of these factors can contribute to the development of early preventive interventions.

Compared with ADHD, relatively few studies have investigated risk factors for comorbid ODD in ADHD. Reported risk factors for ODD, which are arguably also implicated in the development of comorbid ODD, include both risk factors overlapping with those reported for ADHD and risk factors specific for ODD. Overlapping risk factors for ODD and ADHD encompass maternal smoking during pregnancy, a family history of ADHD or ODD, and higher levels of family conflict [6, 36, 39]. Specific risk factors for ODD, compared with ADHD, include deviant peer affiliation, harsh or inconsistent parenting, low levels of parental affection, and exposure to family violence [6, 36, 43]. Studies into specific risk factors for comorbid ODD in ADHD have mainly focused on transgenerational influences, such as parental psychopathology and parenting styles, and reported significant associations of those factors with ODD, rather than with ADHD [for reviews see: 23, 37]. The relative paucity of studies that investigated other environmental risk factors for the development of comorbid ODD in individuals with ADHD is remarkable, given the high prevalence of this comorbid condition.

The aim of the current study was to investigate potential risk factors for the development of comorbid ODD in individuals with ADHD (ADHD + ODD), and to identify whether risk factors differed for individuals with ADHD-only and individuals with ADHD + ODD. To this end, we assessed pre- and perinatal risk factors (pregnancy duration, birth weight, maternal smoking during pregnancy), transgenerational influences (parental ADHD, for ADHD-only and ADHD + ODD parental warmth and parental criticism as well), and postnatal risk factors (socioeconomic status [SES], adverse life events, deviant peer affiliations) in three groups: ADHD + ODD, ADHD-only, and typically developing controls. All groups were matched for age and gender, and the diagnostic groups were additionally matched for IQ and ADHD-subtype. We hypothesised that for both ADHD + ODD and ADHD-only (compared with controls), pre- and perinatal adversities and negative transgenerational influences would be risk factors [6, 33]. In differentiating between ADHD + ODD and ADHD-only, we hypothesised that postnatal risk factors would be more strongly related to ADHD + ODD [1, 23]. Finally, we expected less parental warmth and more parental criticism to be predictive for ADHD + ODD group membership, compared with ADHD-only [23, 43].

## Methods

### Participants

Participants (*N* = 246) were equally divided over three groups: (1) participants with ADHD + ODD (*n* = 82), (2) participants with ADHD-only (*n* = 82), and (3) typically developing controls (*n* = 82). Groups were carefully matched on age (≤1 year) and gender, and the diagnostic groups were additionally matched on IQ [≤10 points, estimated using the Block Design and Vocabulary subtests of the WISC (participants <17) or WAIS (participants 17 and older)] and ADHD-subtype. Mean age of the participants was 16 years (SD 3.1), and each group consisted of 55 boys and 27 girls. See Table 1 for further group characteristics.

**Table 1:** Group characteristics final sample

	ADHD + ODD		ADHD-only		TD		Group comparisons
	(*n* = 45)		(*n* = 45)		(*n* = 42)		
Measure	*M*	SD	*M*	SD	*M*	SD	
Age (years)	16.7	2.7	16.8	2.6	16.3	3.1	ns
IQ	97.2	11.7	97.6	12.4	98.9	8.3	ns
Gender (% Male)	64		64		64		ns
ADHD-type (I/HI/C)	15/3/27	–	15/3/27	–	–	–	ns
ADHD total symptoms^a^	14.2	2.8	13.9	3.0	0.5	1.0	ADHD, ADHD + ODD > TD***
Hyperactive symptoms^a^	6.2	2.2	6.3	2.3	0.2	0.6	ADHD, ADHD + ODD > TD***
Inattentive symptoms^a^	8.0	1.1	7.6	1.6	0.3	0.7	ADHD, ADHD + ODD > TD***
ODD symptoms^b^	5.4	1.2	0.4	0.9	0.0	0.0	ADHD + ODD > ADHD, TD***; ADHD > TD*
CD symptoms^b^	1.0	1.4	0.2	0.5	0.0	0.0	ADHD + ODD > TD***; ADHD + ODD > ADHD**; ADHD > TD*

*ADHD* attention-deficit hyperactivity deficit, *ODD* oppositional defiant disorder, *TD* typically developing, *I* predominantly inattentive type, *HI* predominantly hyperactive-impulsive type, *C* combined type, *ns* not significant

^a^As measured using the combination of K-SADS-PL and Conners scales total, inattentive, hyperactive/impulsive

^b^As measured using the K-SADS-PL

* *p* < .05, ** *p* < .01, *** *p* < .001

Participants were selected from the NeuroIMAGE cohort [55], which included both ADHD families and control families. ADHD families consisted of participants in the ADHD-only or ADHD + ODD group and their biological brothers or sisters, control families consisted of participants in the control group and their biological brothers or sisters. For an overview of the collected data and associated time points, see also the online supplement S1. Inclusion criteria for the current study were: European Caucasian descent, IQ ≥ 80, no diagnosis of conduct disorder, autism, anxiety disorder, depression, epilepsy, general learning difficulties, neurological disorders or known genetic disorders (e.g. Fragile X syndrome, Down syndrome). Individuals in the ADHD + ODD group were only allowed to have an ADHD diagnosis and comorbid ODD, whereas individuals in the ADHD-only group were only allowed to have an ADHD diagnosis. Controls and their first- and second-degree relatives were not allowed to have a past or current DSM-IV diagnosis. A total of 1069 participants (751 children from ADHD families; 318 children from control families) contributed data to NeuroIMAGE, of which 82 participants were diagnosed with both ADHD and ODD and met our inclusion criteria. Individuals with ADHD-only and controls were matched to this group.

### Diagnostic assessment

To determine ADHD and ODD diagnoses, participants were assessed using a combination of the Schedule for Affective Disorders and Schizophrenia for School-Age Children-Present and Lifetime Version (K-SADS-PL) and Conners ADHD questionnaires from multiple informants. For each individual the K-SADS interview was completed by the parent(s), and for individuals aged 12 and older the K-SADS interview was also completed by the participant. Furthermore, each individual was assessed with a teacher-rating (Conners Teacher Rating Scale-Revised:Long version [CTRS-R:L]; 20; applied for participants <18 years) or a Self-Report questionnaire (Conners Adult ADHD Rating Scales-Self-Report:Long Version [CAARS-S:L]; 19; applied for participants ≥18 years). The CTRS-R:L assesses both ADHD and ODD symptoms, while the CAARS-S:L assesses only ADHD symptoms. This combination of assessments ensured that for both ADHD and ODD multi-informant assessments were available: for individuals <12 years old, a parental K-SADS was combined with the CTRS, for individuals aged 12–18, a parental and self-report K-SADS were combined with the CTRS, and for individuals ≥18, a parental and self-report K-SADS were combined with the CAARS. For participants using medication, ratings were done of functioning off medication.

For ADHD, a diagnostic algorithm was applied to combine symptom counts on the K-SADS and CTRS-R:L (for participants <18 years) or CAARS-S:L (for participants ≥18), both providing operational definitions of ADHD defined by the DSM-IV [3]. Participants with ADHD were required to obtain a combined symptom count of ≥6 symptoms of hyperactive/impulsive behaviour and/or inattentive behaviour, provided they: (a) met the DSM-IV criteria for pervasiveness and impact of the disorder (K-SADS), (b) showed an age of onset before 12 (K-SADS), and (c) received a *T* ≥ 63 on at least one of the DSM ADHD scales (total, inattentive behaviour, hyperactive/impulsive behaviour) on either one of the Conners questionnaires [9]. Likewise, for ODD, a diagnostic algorithm was applied to combine symptom counts on the K-SADS and CTRS-R:L (for participants <18 years), both providing operational definitions of ODD defined by the DSM-IV [3]. Participants with ODD were required to obtain a combined symptom count of ≥4 symptoms of oppositional behaviour, provided they: (a) met the DSM-IV criteria for pervasiveness and impact of the disorder (K-SADS), and (b) received a *T* ≥ 63 on the DSM Oppositional behaviour scale of the CTRS-R:L.

### Risk factors

#### Pre- and perinatal factors

Pre- and perinatal risk factors were assessed using a parent-reported questionnaire and included pregnancy duration (weeks) and birth weight (grams). Furthermore, maternal smoking during pregnancy was assessed per trimester and dosage. In the control group, 10 mothers smoked during pregnancy: 2 mothers smoked 11–15 cigarettes per day, 2 mothers smoked 6–10 cigarettes per day, 6 mothers smoked 1–5 cigarettes per day. In the ADHD-only group, 14 mothers smoked during pregnancy: 6 mothers smoked 6–10 cigarettes per day, 8 mothers smoked 1–5 cigarettes per day. Finally, in the ADHD + ODD group, 9 mothers smoked during pregnancy: 2 mothers smoked 11–15 cigarettes per day, 2 mothers smoked 6–10 cigarettes per day, 5 mothers smoked 1–5 cigarettes per day. Given that the number of smoking mothers and the range in dosage was very low together with our findings that our regression models substantially improved when using a dichotomous (yes/no) measure of smoking, rather than a trimester or dosage-related measure, we decided to include the dichotomous measure. Hence, maternal smoking during pregnancy was scored as a ‘yes’ when the mother smoked during at least one trimester.

#### Parental ADHD

Parents were assessed for ADHD, using a similar combination of a semi-structured interview (K-SADS) and an Observer-Rated Symptom questionnaire (Conners) to the one used for the participants. Parental ADHD was scored present if one or both of the parents had a childhood or current diagnosis of ADHD, otherwise it was scored absent.

#### Adverse life events

Parents completed the Long-Term Difficulties questionnaire [developed by TRAILS: 40], which contained 13 items measuring adversities experienced during childhood in multiple settings. These childhood adversities included being bullied, having conflicts with relatives, or relatives having ongoing conflicts among each other, and other persisting problems at home or school such as living in an unsafe neighbourhood. The dependent variable was the total number of adversities the participant had experienced.

#### Socioeconomic status

Socioeconomic status (SES) was assessed using the highest successfully completed educational level of the parents as reported on a Self-Report questionnaire (averaged over both parents). Because in the Netherlands many different trajectories can lead to higher education, it is possible that individuals with a similar amount of educational years differ in their level of education (e.g. senior secondary vocational and pre-university both take 12 years to achieve) [13]. Therefore, we chose, in line with specific studies on this matter in the Dutch society, to recode the highest successfully completed educational level into a measure reflecting years of education, corrected for the level of education [13].

#### Deviant peer affiliation

Deviant peer affiliation was measured using the Friends Inventory, in which participants were asked about the characteristics of their peers (18 items, e.g. ‘my friends break the rules’) [56]. This questionnaire yielded a deviant peer affiliation score (based on nine items) with higher scores indicating less deviant peers. Good internal consistency [.78–.92, see: 16, 31, 56], and inter-rater reliability [.71, see: 31] have been reported.

#### Expressed emotions (parental warmth and criticism)

Expressed emotions (EE) was assessed during the diagnostic interview (PACS) of the IMAGE-study, which was performed 6 years previous to the current study [49], using the scoring derived from Camberwell Family Interview [12]. Scores from both parents were averaged. Warmth was assessed by the tone of voice, spontaneity, sympathy, and/or empathy toward the child (range 0–3). Criticism was assessed by statements which criticised or found fault with the child based on tone of voice and critical phrases (range 0–4) [44, 49]. Inter-rater reliability has been found adequate for warmth and criticism (range .78–91 and .79–.86, respectively; [46]). An average agreement percentage of 96.6% (range 78.6–100) and a mean Kappa coefficient of .88 (range .71–1.00) have been reported [38]. Data were available for the diagnostic groups only.

### Procedure

The current study was part of a comprehensive assessment protocol encompassing phenotypic, neurocognitive, and magnetic resonance imaging assessments [55]. Data on risk factors were assessed for all groups, except for parental warmth and parental criticism, which were only available for the diagnostic groups. Informed consent was signed by all participants (for participants <12 years only parents signed informed consent, for participants between 12 and 18 years both the participants and their parents signed, for participants >18 years only the participants signed), and the study was approved by the local ethics committees.

### Statistical analyses

All dependent variables were normally distributed and did not contain outliers (defined as a score of >3 SD from the mean score). Groups were compared on demographic and ADHD-related variables using analysis of variance (ANOVA) or Chi-square tests. For pre- and perinatal risk factors, data were available for 57–81% of the subjects. For other risk factors, there were some missing data (20.7% deviant peers, 6.1% parental warmth, 4.1% SES, 1.6% adverse life events), which were mainly due to not assessed questionnaires, logistic problems or incompletely filled out questionnaire, and were randomly distributed over the groups. The assessed analysis (LASSO; see below) does not allow missing data on the predictors; therefore, cases with missing values were omitted from the analysis. Since LASSO can be applied even in models with the number of predictors exceeding the number of participants, the smaller sample size poses no problem in the current study where the number of participants still substantially exceeds the number of predictors. Final sample size was *N* = 86 participants for the analyses for controls versus ADHD-only and for the analyses for controls versus ADHD + ODD, while for ADHD-only versus ADHD + ODD the final sample size was *N* = 90.

Due to the amount of missing data, we investigated whether the resulting subsamples differed from the initial matched samples. Results of these comparisons showed that the subsamples were still matched for age, gender, IQ, and ADHD-type (for diagnostic groups), and showed similar group comparison results in terms of ADHD symptom and ODD symptom levels, as shown in Table 1.

For the predictive value analysis of the complete set of risk factors, least absolute shrinkage and selection operator (LASSO) penalised logistic regression in R was used, using the *glmnet*-package [51]. The LASSO approach is a shrinkage and selection method for logistic regression with the advantage of automatically assigning a penalized term to the predictors, and thereby selecting a model with the best fitting set of predictors [51, 52]. That is, LASSO selects an optimal set of predictors that is a trade-off between the number of predictors and the amount of explained variance. The outcome measure was group membership, being either control group, ADHD-only group, or ADHD + ODD group. Selection of the strength of the penalty term was performed through cross-validation (20-fold). For this model, the percentage of explained deviance was calculated, which compares to the explained variance (i.e. the deviance is the increase in explained variance over the explained variance of the null (intercept-only) model) [51]. Post hoc, an estimation of the explained deviance for each selected predictor was calculated by selectively leaving out one predictor and re-running the analysis. Since the explained deviance of the model is based on the total set of predictors, the estimations for the single predictors deviate from the total explained deviance due to combined effects of predictors and the estimation routine. Therefore, we also report the beta coefficients for the single predictors. In addition, interactions between each of the predictors in the model and both age and age^2^ were investigated. Finally, to control for the possible impact of family relations, due to the inclusion of siblings, we performed sensitivity analysis excluding the siblings (20 controls, 2 ADHD-only, 2 ADHD + ODD).

The analyses comprised three models to identify the risk factors for comorbid ODD in ADHD. First, two separate models were assessed to investigate risk factors for ADHD-only compared with controls (model 1) and risk factors for ADHD + ODD compared with controls (model 2). Subsequently, the model for the ADHD + ODD group versus the ADHD-only group (model 3) was assessed. Significance of a risk factor was assumed if the LASSO model included that factor. Finally, for each of the three models, the sensitivity (percentage correctly identified cases) and specificity (percentage correctly identified non-cases) were calculated.

## Results

As shown in Table 1, the diagnostic groups did not differ in number of ADHD total, hyperactive/impulsive or inattentive symptoms, measured using the combination of the K-SADS and Conners questionnaires. There were no group differences in IQ between any of the groups. As a result of our matching procedure, groups did not differ on age and gender, and the diagnostic groups did not differ on ADHD-subtype. Table 2 shows the predictor characteristics for the three groups.

**Table 2:** Predictor characteristics per group

Measure	ADHD + ODD		ADHD-only		TD	
	*M*	SD	*M*	SD	*M*	SD
Parental ADHD (%)	55.3	–	68.2	–	0	–
Adverse life events	2.8	2.7	1.8	1.7	0.6	0.8
Birth weight (gram)	3427.4	642.5	3588.4	510.9	3360.7	698.3
Pregnancy duration (weeks)	39.3	2.4	39.4	2.0	39.3	2.2
Maternal smoking during pregnancy (%)	21.6	–	27.9	–	19.0	–
SES (corrected years of education)	11.0	1.7	11.7	2.1	12.9	2.6
Deviant peer affiliation	27.4	5.0	29.8	5.6	31.4	3.5
Parental warmth	1.7	0.9	1.4	0.8	–	–
Parental criticism	1.9	0.9	1.7	0.9	–	–

*ADHD* attention-deficit hyperactivity deficit, *ODD* oppositional defiant disorder, *TD* typically developing, *SES* socioeconomic status

Results for all three models are shown in Table 3, with the total explained deviance per model, and estimation of the explained deviance and beta coefficient per predictor. There were no significant interactions between any of the predictors and age, age^2^ or age^3^ (*p* > .105), indicating that the effects of the predictors on diagnostic status were independent of the age of the participants.

**Table 3:** Explained deviance and beta coefficients

	TDC versus ADHD-only		TDC versus ADHD + ODD		ADHD-only versus ADHD + ODD	
	Explained deviance (%)	Beta coefficient	Explained deviance (%)	Beta coefficient	Explained deviance (%)	Beta coefficient
Total model	58.4		62.5		15.3	
Parental ADHD	27.2	3.91	21.9	3.34	2.4	−0.42
Adverse life events	6.0	0.50	13.9	0.57	3.0	0.12
Birth weight	*ns*	0.00	2.0	0.00	*ns*	0.00
Pregnancy duration	*ns*	0.00	*ns*	0.00	*ns*	0.00
Maternal smoking during pregnancy	1.3	0.00	*ns*	0.00	*ns*	0.00
Socioeconomic status	*ns*	0.00	1.5	−0.16	5.6	−0.18
Deviant peer affiliation	*ns*	0.00	3.8	−0.15	4.8	−0.05
Parental warmth	–	–	–	–	*ns*	0.00
Parental criticism	–	–	–	–	6.4	0.34

Percentage per predictor is an estimation, hence the separate percentages do not add up to total model. Parental warmth and parental criticism scores were only available for diagnostic groups and, therefore, not included in models assessing predictors against controls

*ADHD* attention-deficit/hyperactivity disorder, *ODD* oppositional defiant disorder,* TDC* typically developing controls, *ns* not significant

### Risk factors for ADHD: ADHD-only versus typically developing controls

Predictors that were initially inserted in the model were pregnancy duration, birth weight, maternal smoking during pregnancy, parental ADHD, adverse life events, SES, and deviant peer affiliation. Correlations between predictors were below *r* = .42. After LASSO selection, the model showed a total explained deviance of 55.2% (see Table 3) and showed that parental ADHD (27.2%), higher levels of adverse life events (6.0%), and maternal smoking during pregnancy (1.3%) were associated with a heightened risk for ADHD-only compared with controls. The model showed a sensitivity of 98% and specificity of 80%.

### Risk factors for ADHD + ODD: ADHD + ODD versus typically developing controls

Predictors initially inserted in the model were identical to those inserted in the model for ADHD-only versus controls. Correlations between predictors were below *r* = .42. After LASSO selection, the total explained deviance was 62.5% (see Table 3) and parental ADHD (21.9%), more adverse life events (13.9%), more deviant peer affiliations (3.8%), higher birth weight (2.0%), and lower SES (1.5%) were associated with a heightened risk for ADHD + ODD compared with controls. The model showed a sensitivity of 95% and specificity of 87%.

### Risk factors for comorbid ODD: ADHD-only versus ADHD + ODD

In addition to the predictors inserted in the previous models, this model also included parental warmth and parental criticism. Correlations between predictors were below *r* = .43. After LASSO selection, the model showed a total explained deviance of 15.3% (see Table 3) and higher levels of parental criticism (6.4%), lower SES (5.6%), deviant peer affiliation (4.8%), more adverse life events (3.0%), and parental ADHD (2.4%) were associated with a heightened risk for comorbid ODD in individuals with ADHD. The model showed a sensitivity of 90% and specificity of 57% for predicting the presence of comorbid ODD.

#### Family-corrected results

For analyses of risk factors for ADHD-only and for ADHD + ODD versus controls, 22 siblings were excluded, whereas for the analysis of risk factors for comorbid ODD, 4 siblings were excluded. For the first two models, the total explained deviance of the model was reduced somewhat (to 51.9 and 57.5%, respectively), while for the comorbid ODD model the explained deviance increased slightly (to 17.6%). All models included the same predictors with the same direction of associations.

## Discussion

The aim of the current study was to investigate risk factors for the development of comorbid ODD along with ADHD. Therefore, we assessed pre- and perinatal factors, transgenerational influences and postnatal factors. We hypothesised that pre- and perinatal adversities and negative transgenerational influences would act as risk factors for both ADHD and ADHD + ODD, and postnatal adversities to act primarily as risk factors for ADHD + ODD compared with ADHD-only [1, 6, 23, 33]. Additionally, in differentiating between ADHD-only and ADHD + ODD, we hypothesised that postnatal adversities and negative transgenerational influences would be more strongly related to ADHD + ODD than to ADHD-only [43]. Our models identified several risk factors for ADHD + ODD and for ADHD-only, compared with controls, with high levels of explained deviance of 55.2 and 62.5%, respectively. Our model for risk factors differentiating between ADHD + ODD and ADHD-only showed an explained deviance of almost 15.3%. All three models showed good sensitivity (90–98%), and the models for the control group versus both the ADHD + ODD and the ADHD-only groups also showed good specificity (80–87%). We found no interaction between age and any of the risk factors, indicating that predictors are equally important during all stages of development in our sample, and independent of age of the participants (age 7–24 years).

Our first hypothesis that negative transgenerational influences and pre- and perinatal adversities would act as risk factors for the diagnostic groups was supported by our findings, since we found that parental ADHD acted as a relatively major risk factor within our models, showing the highest explained deviance for both diagnostic groups relative to the control group. This is in line with the many studies showing significant heritability rates for ADHD [10, 22], and large effects of environmental influences associated with parental ADHD on the development of ADHD in the child [8]. In terms of pre- and perinatal adversities, maternal smoking during pregnancy acted as a relatively minor risk factor within our model for ADHD-only, while *higher* (not lower) birth weight acted as a relatively minor risk factor for ADHD + ODD, relative to controls. This supports the notion that there may be an optimum birth weight in terms of the development of behavioural problems such as ADHD, as previously suggested in other studies [17, 25, 54]. We were not able to replicate previously reported findings of lower birth weight or pregnancy duration as risk factors for ADHD [50], which may be due to the small number of individuals with a low birth weight or premature birth in our sample (8 and 12, respectively). To conclude, our findings show parental ADHD as a significant risk factor and suggest that the relationship between birth weight and pregnancy duration and the development of ADHD might only hold true for values below a certain threshold.

We also found support for our second hypothesis of postnatal adversities acting as risk factors for ADHD + ODD rather than for ADHD-only. For both ADHD-only and ADHD + ODD, adverse life events, which included parental divorce and family conflicts, acted as a risk factor. However, adverse life events acted as a stronger risk factor for ADHD + ODD than for ADHD-only, as stressed by its differentiating ability between the ADHD + ODD and ADHD-only groups. The mechanism by which adverse life events may affect ODD is still unclear, and may vary between types of event; potential explanations include (a) negative effects on maturation of cerebral brain structures in the child due to stress, (b) teaching individuals to use antisocial strategies to cope with stressful situations, and (c) causing an overactive sympathetic nervous system [7, 29]. All these factors have been implicated in the development of ODD and receive extensive support, suggesting a combination of these risk factors to operate in ODD [7, 29]. While no other risk factors were observed for the development of ADHD-only, more deviant peer affiliations and lower SES did act as additional risk factors for ADHD + ODD, compared with controls. This is consistent with previous studies showing that more deviant peer affiliations reinforce an individual’s own antisocial behaviours [18, 30, 48]. SES acted as a relatively minor risk factor within our model (1.5–5.6%), presumably exerting its effect through poor parenting and deviant socialisation processes that are associated with lower parental SES [42]. The relatively weak effect of SES may be due to its relationship to parental ADHD, which was also included in the model (e.g. lower parental mental health has been associated with lower SES) [45]. Since both deviant peer affiliations and SES differentiated between ADHD + ODD and ADHD-only, these risk factors seem especially important for the development of comorbid ODD.

Our third hypothesis, that transgenerational influences in addition to the postnatal factors would differentiate between ADHD + ODD and ADHD-only, was largely supported by our results. Parental criticism acted as a relatively strong risk factor for ADHD + ODD compared with ADHD-only, within our model. This is in line with previous studies and is presumably due to its negative influence on the child’s socialisation process [8, 23, 37]. In addition, it has been reported that child difficulty not only increases the likelihood of maternal negative parenting, but also that maternal negative parenting heightens the child’s behavioural maladjustment that may take the form of ODD behaviours [11]. This is in line with the coercion theory that describes a process of mutual reinforcement between the parent and child in the development of conduct problems. According to this model the parent inadvertently reinforces the child’s difficult behaviour by reacting negatively to that behaviour and therewith escalating the situation [47]. Hence, negative parental attitudes are risk factors not only for ADHD [23], but especially for comorbid ODD. Furthermore, when parents express low levels of support, the negative influences of deviant peers on the development of oppositional behaviour increase [26, 53]. Against our hypothesis and contradicting previous studies, parental ADHD acted as a minor protective factor for the development of comorbid ODD in ADHD [23]. A possible explanation may be that children with comorbid ODD are more difficult to handle, thereby increasing the likelihood of parents to seek professional help. However, this remains speculative and requires further investigation.

Even though our study has some important strengths, there are some limitations too. First, we assessed pregnancy duration, birth weight and maternal smoking during pregnancy using a retrospective parent questionnaire. Especially for maternal smoking during pregnancy, the self-report nature of our assessment may have confounded our data, due to socially acceptable answering [24]. However, we did only investigate whether or not the mother smoked, excluding dosage effects and thereby limiting the influence of socially acceptable answering (such as reporting lower dosages). Moreover, there was a relatively large amount of missing data for pre- and perinatal information. Second, even though we assessed parental ADHD and parental psychopathology, we did not specifically assess paternal antisocial personality disorder or maternal stress, which both have been found to be related to the development of antisocial behaviour disorders [33]. Further, we did not assess parenting styles, which would have allowed us to investigate further the alleged link between parental ADHD and deviant parenting styles. In addition, parental criticism and warmth were only assessed in the diagnostic group, limiting our findings to the diagnostic groups comparison, and thus to predictors for comorbid ODD versus ADHD-only. Third, even though we assessed robust prediction models, our findings are based on a combination of longitudinal (parental warmth and criticism), retrospective (birth weight, pregnancy duration, maternal smoking during pregnancy, adverse life events, parental ADHD, parental SES), and cross-sectional (deviant peer affiliations) data. However, the retrospective predictors were independent of the measurement period, and only deviant peer affiliation data were assessed cross-sectional. For the latter variable, our final model may have been different if it had been measured at baseline. However, although it has been suggested that the influence of deviant peer affiliations would change over development, the level of deviant peer affiliations appears to be stable over development [28]. Fourth, especially for the control group, a relatively large proportion of siblings was included. Nevertheless, our findings did not change in terms of the predictors involved or the direction of associations when excluding these siblings, indicating that the findings are robust. Finally, since we applied strict inclusion criteria, such as excluding individuals with a comorbid conduct disorder diagnosis, our sample may represent a subsample of, rather than all, individuals with ADHD-only and ADHD + ODD. In addition, we focused on comorbid ADHD + ODD and were not able to include an ODD-only group. Therefore, the findings may not be generalizable to all individuals with ADHD or individuals with only ODD.

Overall, our study showed that postnatal risk factors (adverse life events) and transgenerational influences (parental ADHD) are important risk factors for the development of ADHD + ODD and ADHD-only. The development of comorbid ODD in individuals with ADHD was predicted by both postnatal adversities (SES, deviant peer affiliation) and negative transgenerational influences (parental criticism). These risk factors were significant for all ages. Our findings are in line with theories stating that environmental factors play an important role in the development of comorbidities such as ODD in individuals with ADHD [5, 23, 50]. This highlights the need to take these risk factors into account when treating children with ADHD, since these factors may prove to be essential in the prevention of comorbid ODD. The development of comorbid ODD in ADHD is of concern given the lower functional outcome of this comorbid group relative to either disorder separately [4, 35]. For example, (comorbid) ODD is reported as an important predictor for later life conduct disorder [15]. Our findings seem to support the use of intervention programs comprising parent- and parent–child training in the prevention of comorbid ODD [34], although we did not assess these trainings or their effects ourselves. In addition, monitoring peer affiliations of individuals with ADHD may prove useful in averting the transition from ADHD-only to the more severe ADHD + ODD [8, 23, 37].

## Electronic supplementary material

Below is the link to the electronic supplementary material.

Supplementary material 1 (DOCX 16 kb)
