# Supplementary material for: Risk factors for comorbid oppositional defiant disorder in attention-deficit/hyperactivity disorder
Source: Eur Child Adolesc Psychiatry. 2017 Mar 10;26(10):1155–64. doi: 10.1007/s00787-017-0972-4 (PMC5610221; doi:10.1007/s00787-017-0972-4)
Supplement: Supplementary file 1 — Supplementary material 1 (DOCX 16 kb) [file 787_2017_972_MOESM1_ESM.docx]

**Supplement S1 - Information on the cohort and assessment of predictors.**

*Adapted from “The NeuroIMAGE study: a prospective phenotypic, cognitive, genetic and MRI study in children with attention-deficit/hyperactivity disorder. Design and descriptives”, by D. Von Rhein et al., 2015. Copyright 2015, adapted with permission.*

**The cohort***Original IMAGE cohort (2003–2006)*Participants for NeuroIMAGE were selected from the Dutch part of the International Multicenter ADHD Genetics (IMAGE) study, conducted between 2003 and 2006. In the Dutch part of IMAGE 365, families with at least one child with combined subtype ADHD and at least one biological sibling (regardless of ADHD diagnosis) were recruited, in addition to 148 control families with at least one child, with no formal or suspected ADHD diagnosis in any of the first degree family members. Inclusion criteria for the IMAGE study were: participants had to be between 5 and 30 years, of European Caucasian descent, have an IQ >70, and no diagnosis of autism, epilepsy, general learning difficulties, brain disorders, and known genetic disorders (such as Fragile X syndrome or Down syndrome).

*NeuroIMAGE (2009–2012)*
For NeuroIMAGE, all family members, including those who did not participate in IMAGE, were invited for follow-up measurement and (re)assessed between 2009 and 2012. The time between the IMAGE and NeuroIMAGE measurements ranged between 3.5 and 8.9 years (overall *M* = 5.9 years, S*D* = 0.74). Additionally, children with ADHD (foremost girls) and healthy control boys were newly recruited to balance the distribution of gender and age between the ADHD and healthy control groups in NeuroIMAGE. Inclusion criteria were largely consistent with the IMAGE study, except that we now allowed inclusion of children with any subtype ADHD rather than the combined subtype only.
 Including the newly recruited families, the complete NeuroIMAGE cohort comprised testing of more than 1,000 children and approximately 850 tested parents. Retention rate from the original IMAGE study was high (79 %). The most important reasons for drop-out were being too busy, family problems, and time consumption of the study.

**Measures**
Data investigated in the current study included both assessments from the IMAGE and the NeuroIMAGE study.

During IMAGE-I, Expressed Emotions (EE) was assessed during the diagnostic interview. EE provided scores of parental warmth and parental criticism. Furthermore, parents filled out a questionnaire on demographic characteristics, including pre- and perinatal information and socioeconomic status.

During NeuroIMAGE, questionnaires compromising several domains of functioning, including adverse life events and deviant peer affiliations were completed. The questionnaire on adverse life events was filled out by the parents of the participant. The questionnaire on deviant peer affiliations was filled out by the participant. For participants younger than 12 years, their parents or the researchers assisted in the completion of the self-report questionnaires. Additionally, assessments for NeuroIMAGE included a semi-structured clinical interview (Dutch translation of the Schedule for Affective disorders Schizophrenia—present and lifetime version (K-SADS)). The K-SADS is designed to assess current and past episodes of psychopathology in children, adolescents, and adults according to DSM-IV criteria.
